# Optimal Design of Bubble Deck Concrete Slabs: Serviceability Limit State

**DOI:** 10.3390/ma16144897

**Published:** 2023-07-08

**Authors:** Tomasz Gajewski, Natalia Staszak, Tomasz Garbowski

**Affiliations:** 1 Institute of Structural Analysis, Poznan University of Technology, Piotrowo 5, 60-965 Poznan, Poland; tomasz.gajewski@put.poznan.pl; 2 Doctoral School, Poznan University of Life Sciences, Wojska Polskiego 28, 60-637 Poznan, Poland; natalia.staszak@up.poznan.pl; 3 Department of Biosystems Engineering, Poznan University of Life Sciences, Wojska Polskiego 50, 60-627 Poznan, Poland

**Keywords:** lightweight structures, bubble deck concrete slabs, numerical homogenization, weight minimization, sequential quadratic programming

## Abstract

In engineering practice, one can often encounter issues related to optimization, where the goal is to minimize material consumption and minimize stresses or deflections of the structure. In most cases, these issues are addressed with finite element analysis software and simple optimization algorithms. However, in the case of optimization of certain structures, it is not so straightforward. An example of such constructions are bubble deck ceilings, where, in order to reduce the dead weight, air cavities are used, which are regularly arranged over the entire surface of the ceiling. In the case of these slabs, the flexural stiffness is not constant in all its cross-sections, which means that the use of structural finite elements (plate or shell) for static calculations is not possible, and therefore, the optimization process becomes more difficult. This paper presents a minimization procedure of the weight of bubble deck slabs using numerical homogenization and sequential quadratic programming with constraints. Homogenization allows for determining the effective stiffnesses of the floor, which in the next step are sequentially corrected by changing the geometrical parameters of the floor and voids in order to achieve the assumed deflection. The presented procedure allows for minimizing the use of material in a quick and effective way by automatically determining the optimal parameters describing the geometry of the bubble deck floor cross-section. For the optimal solution, the concrete weight of the bubble deck slab was reduced by about 23% in reference to the initial design, and the serviceability limit state was met.

## 1. Introduction

Over the last few decades, concrete structures, in particular prefabricated reinforced concrete structures, have gained popularity and found wide application in many construction sectors around the world [1]. They are used not only in industrial, commercial, or residential facilities, but also in infrastructural construction. Prefabricated reinforced concrete elements, among others, mainly include columns, foundation footings, and retaining walls, as well as prefabricated walls with window and door openings. However, the most commonly used prefabricated concrete elements are girders and floor slabs [2].

This type of construction has numerous advantages, including: (a) saving formwork, (b) high durability and resistance of the structure, (c) high strength, (d) short construction time, (e) quality standards, and (f) reducing the amount of work on construction sites. In addition, prefabricated structures can be shaped in many ways using modern technologies and adapted to local conditions that occur in the designed facilities. Moreover, during the production stage, it is possible to make cuts and openings which allow for carrying out installation, e.g., pipes or cables after mounting the element at the site. It is extremely important to have already planned and anticipated all required openings in the design stage. On the other hand, prefabricated elements also have disadvantages. One of the main disadvantages is their cost, which consists of production, transport, and the need for cranes for their assembly [3]. However, the latter is not a problem, because on large construction sites, in which such structures are used, heavy equipment is available to unload and assemble them.

The main idea of the floor technology has remained unchanged in its general concept for years. Each type of ceiling should meet specific requirements that determine the selection of the appropriate structure and technology [4]. Technical requirements, including thermal and acoustic insulation, adequate strength, stiffness, fire resistance, and durability, have the greatest impact. Another important aspect is economic requirements, which include minimization of costs during construction and the project design stage. In the case of large spans between supports, prefabricated floors are used. The basic types of such floors are solid slabs, filigree slabs, multi-hole slabs, and TT double-rib slabs [5]. In such structures, an important aspect is to increase the span, and at the same time, reduce the weight of the panels [6]. Therefore, lightweight concrete structures are increasingly used, e.g., channel slabs or bubble deck slabs. This approach aims to eliminate the concrete that does not fulfill any structural functions to reduce the weight, and thus, the dead load [7,8].

Over the last few decades, many numerical models have been developed to represent the global behavior of prefabricated concrete structures and to understand their mechanical behavior. These models serve as valuable tools for simulating and analyzing the structural response of prefabricated elements, enabling engineers to evaluate their performance under various loading conditions and optimize their design. For instance, the authors of [9] presented a full 3D model of prefabricated bridge slabs for the purpose of modeling their non-linear behavior using the constitutive model of concrete damage plasticity. In turn, the bending behavior of composite slabs was analyzed by Tzaros et al. [10]. Gholamhossein et al. [11] proposed a three-dimensional solid finite element model to investigate the connection between concrete and steel. Information regarding the puncture resistance of concrete slabs can be found in [12]. Using the finite element method (FEM), it becomes possible to analyze structures with complex shapes and geometries, and to gain insight into the non-linear behavior of concrete and steel [13,14,15]. However, the detailed modeling of three-dimensional prefabricated slabs requires a lot of work, specialist knowledge, as well as the use of specific software, and it is very time-consuming in terms of calculations. The solution to the problem may be the use of one of the homogenization methods.

Homogenization is a mathematical technique used to analyze and model the behavior of heterogeneous materials or structures. It aims to capture the effective properties of an entire material or structure, taking into account its constituent materials or components. In the context of construction and structural calculations, homogenization refers to the simplification or adoption of uniform material or structure properties in order to facilitate calculations. For materials with an irregular structure or composition, simplifications can be used, where the material is treated as having uniform properties such as strength, stiffness, and density. Homogenization can also refer to simplification in structural analysis, where complex models of structural elements or details are replaced by simpler models that account for similar structural behavior. One can distinguish, among others, the method of periodic homogenization [16], non-linear homogenization [17], or the method of multi-scale homogenization based on genome mechanics [18]. A slightly different approach can be found in the work of Garbowski and Marek [19], where a method based on reverse analysis was used. On the other hand, in [20], a method of homogenization based on strain energy for sandwich panels with a honeycomb core was presented. Furthermore, Biancolini developed a method of strain energy equivalence between the simplified model and the representative volumetric element (RVE) model [21]. This method was then extended in [22]. Homogenization may introduce some simplifications and approximations, so its use should be carefully assessed in the context of a specific project and the fulfillment of relevant load-bearing and safety requirements.

Homogenization methods are also used in structural optimization analyses. This refers to the process of designing and modifying a building or structural elements to achieve the best possible results in terms of strength, cost-effectiveness, and energy efficiency. In addition, structural optimization is a complex process that requires consideration of multiple parameters and various engineering disciplines. It includes the integration of knowledge from structural engineering, materials science, mechanical engineering, and other relevant fields. The goal of the optimization is to find the best compromise between individual design requirements, so that the construction is as efficient, durable, and economical as possible, taking into account factors such as structural loads, material properties, construction techniques, and environmental impacts. This process may include the analysis of various scenarios, e.g., changing the geometry, materials, and configuration of structural elements to find the most suitable solution. The use of advanced tools such as structural analysis software and computer simulations can greatly facilitate the optimization process and help in obtaining optimal design solutions. These tools allow engineers to model and simulate the behavior of the structure under various conditions, accurately predict its performance, and evaluate different design alternatives.

Many studies have been conducted that provide information on the optimization of building construction, e.g., prefabricated elements, steel, or wooden structures. Sotiropoulos et al. presented a conceptual design method based on topology optimization using prefabricated structural elements [23]. The hybrid optimization method was used to optimize cellular beams in [24]. The optimization of thin-walled cross-sections was shown in [25]. Furthermore, the work by Sojobi et al. [26] presented a multi-objective optimization of a prefabricated carbon fiber-reinforced polymer (CFRP) composite sandwich structure. Additionally, Xiao and Bhola [27] presented the design of prefabricated building systems using building information modeling (BIM) technology and structural optimization. In the work by Xie et al. [28], a genetic algorithm was utilized for optimal planning of prefabricated construction projects.

In addition, many studies have been performed on the plates analyzed in this publication. Abishek and Iyappan investigated the bending behavior of a reinforced FRP (fiber-reinforced polymer) bubble deck [29]. Additionally, in [30], the evaluation of the plasticity of reinforced concrete structures with voids was analyzed. Experimental testing of slabs with modified openings was discussed in [31], and the study of concrete using plastic waste applied to hollow plates was presented in [32]. John et al. [33] demonstrated the bubble deck behavior using a model in ANSYS. They analyzed service load deflection, crack pattern, concrete stress distribution, and ultimate load capacity.

In our previous paper [34], a sensitivity study regarding the main bubble deck parameters was performed, i.e., it was checked how the changes of a single engineering parameter (such as bubble dimensions, class of concrete, reinforcement diameter, number of bars, etc.) influence the particular effective stiffnesses of the BD230 and BD340 slabs. In the current paper, the optimal design for a particular slab is sought by employing the minimization technique. According to the prior state-of-the-art review, there are no studies on the optimal design of the bubble deck plate, although this slab technology has many advantages. Determining the optimal design of the bubble deck slab through the numerical homogenization technique is the main novelty of the paper.

This paper presents an algorithm for the optimal design of bubble deck construction in order to minimize the amount of concrete and ensure that the permissible deflection arrow of the structure in the serviceability limit state is not exceeded. To simplify the model and speed up the analysis, the numerical homogenization method based on the equivalence of the strain energy between the simplified shell model and the three-dimensional reference RVE bubble deck model was used. The analyzed concrete slab contains evenly distributed voids over the entire surface and both upper and lower steel reinforcement, which increases the complexity and time of the calculations. Therefore, the original numerical homogenization method, initially developed for shell structures, was modified. In the work, an extension of the method was used to simultaneously include continuum and truss elements, similar to in [34,35].

One of the most important limitations of this study is that currently, the stress analysis cannot be included while using the homogenization technique considered [21,22]. Therefore, the ultimate limit state is beyond the scope of the current study. Another limitation is that the results from optimization with respect to the parameters of continuous domains may not be straightforward to be implemented in the industry. The use of concrete will be less in the optimal solution, but it is likely that bubble deck voids will be more expensive than the traditional bubble deck solution.

## 2. Materials and Methods

### 2.1. Lightweight Concrete Slabs

#### 2.1.1. Voided Floor Slabs

The expression “voided floor slabs” refers to a special type of construction where the concrete slab contains a system of regular hollows or empty spaces inside. The hollows may have various shapes, such as spheres, cylinders, clovers, etc. In addition, they can be arranged in a specific pattern or grid. Voided floor slabs are used to achieve greater efficiency in terms of material use and to reduce the weight on the structure, while ensuring the required strength and performance of the floor structure. One of the most common types of voided floor slabs is the bubble-type ceiling.

The bubble deck is a modern ceiling solution compared to traditional slabs, in which the ineffective concrete in the middle of the cross-section is replaced by voids. This floor slab was invented in the 1990s by Danish engineer Jorgen Breuning [36]. It is used in residential, office, industrial, and utility buildings. In addition, the bubble deck ceiling is used in factories, parking structures, schools, and hotels.

Bubble deck has spherical or elliptical voids [37] evenly distributed over the entire surface, without changing the two-way action of the element. This solution allows to reduce the amount of concrete by 33% and the price by 30% compared to traditional solid slabs with the same parameters [38]. One of the main differences between solid slabs and bubble deck is their shear resistance [39]. In addition, by adjusting the appropriate reinforcement mesh and hole geometry, an optimized concrete structure can be achieved, allowing for the simultaneous maximum utilization of both moment and shear zones.

The height of such slabs can achieve up to 600 mm, which allows for a span of up to 20 m. On the other hand, the diameter of the bubble made of HDPE (high-density polyethylene) varies between 180 and 450 mm, depending on the designed thickness of the slab. The HDPE material used comes from recycled plastic waste. Therefore, the above solution contributes to the reduction of pollution and has a positive impact on environmental protection. However, the distance between the individual voids in the bubble deck system must be at least 1/9 of the bubble diameter. This requirement ensures adequate structural integrity and optimal load distribution in the floor slab. By keeping the distance between voids to a minimum, the system can effectively reduce the weight of the structure, while providing sufficient strength and stability. The voids are fixed in special steel baskets between the upper and lower reinforcements of the plate to prevent their displacement during concrete pouring [40,41]. In addition, the slab is directly connected to concrete columns or walls in situ without any beams, providing a wide range of structural costs and benefits. This eliminates the need for additional structural elements, simplifies the construction process, and allows for more flexible design options. Such a direct connection between the slab and columns or walls enhances the structural efficiency and optimizes the construction costs.

**Figure 1:**
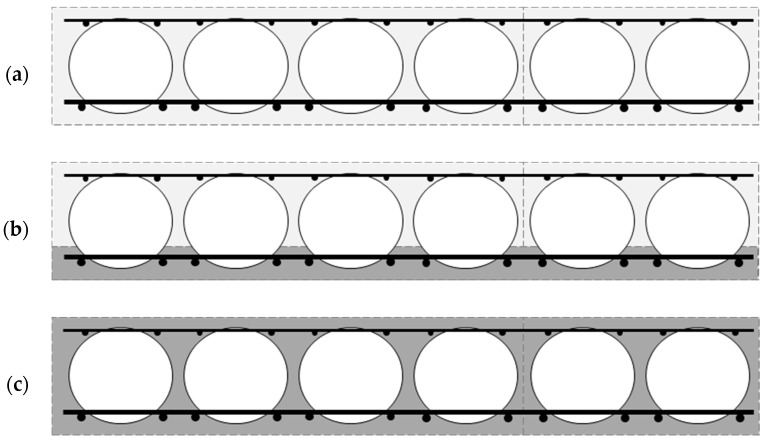
Types of bubble deck: (**a**) in situ element, (**b**) semi-prefabricated element, and (**c**) fully prefabricated element.

There are three methods of manufacturing the bubble deck: (i) the in situ approach—Figure 1a, (ii) semi-prefabricated elements (Filigree element)—Figure 1b, and (iii) prefabricated panels—Figure 1c [42]. In the case of the in situ approach (i), the slabs are made onsite at the place of their installation. Voids are placed in specific places between the bottom and top reinforcements of the floor. In the next step, the finished modules are placed on the prepared formwork. Then, the slab is concreted. It is worth noting that this solution is very effective in buildings where the floor is not flat, e.g., there is a domed or curved ceiling. In the case of approach (ii), the deck is a semi-prefabricated bubble deck. This means that elements are created in the production plant, which then require additional concreting at the construction site. This is a combination of methods (i) and (iii). The lower part of the ceiling, which is also the permanent formwork, is in the form of a concrete slab with a thickness of approximately 6 cm. In addition, it has both lower and upper reinforcements, with spaced voids. Then, the whole element is transported to the construction site and concreted after moving to the appropriate place. In contrast, approach (iii) deals with components that are fully manufactured. The finished prefabricated elements are transported to the construction site. This solution partially limits the ability of the plates to function as bi-directional. The solution to this problem may be the proper design of the connection between the prefabricated slabs, which will enable the use of prefabricated elements as two-way floors, similar to approaches (i) and (ii).

The bubble deck is generally designed using conventional solid-ceiling design methods, in accordance with applicable international and local design standards. The above floor system is a true bidirectional monolithic slab and behaves as a solid slab in both elastic and plastic modes. This means it can effectively carry and distribute loads in a manner similar to traditional solid slabs, ensuring structural integrity and performance. Besides, it mainly uses two analysis methods in its design, such as the linear elastic and yield line methods. Thanks to the optimized geometry and spherical bubbles, every part of the concrete in the slab is actively involved and relevant in the calculation of different types of forces.

#### 2.1.2. Serviceability Limit State of Bubble Deck Concrete Slabs

During the design and analysis of structures, it is important to check two main limit states to ensure the safety and strength of the structure: the ultimate limit state (ULS) and the serviceability limit state (SLS). The first one concerns the assessment of the load capacity of the structure and checking whether it is able to withstand loads, in accordance with the adopted standards, building regulations, and design requirements. For different types of structures, such as floors, columns, foundations, bridges, etc., the ULS refers to the evaluation of their strength in response to various forces, such as compression, bending, tension, bending moments, shear forces, etc. The strength of the bubble deck is typically determined by structural analysis and calculation, taking into account factors such as the flexural strength, shear capacity, and the ability to efficiently distribute loads.

The serviceability limit state (SLS) refers to the conditions in which the structure can be operated without unacceptable deformations or damage that may affect its functionality and safety. In the case of bubble boards, the key aspect is to ensure that the bubbles inside the board are stable and do not undergo deformation or damage that could affect the load capacity and stiffness of the board. The SLS covers various aspects, including deflections and cracks. In the case of slabs, there are certain permissible deflection limits that should not be exceeded to ensure the stability and functionality of the structure. Excessive deflections can lead to improper functioning of finishing elements, problems with water drainage, or deterioration of interior aesthetics. Usually, when assessing the SLS, it is recommended to examine the cracks to determine their nature, size, and their impact on the safety and functionality of the structure.

In this work, analyses of the optimization of the bubble deck ceiling in terms of the SLS were carried out. It was assumed that the maximum plate deflection cannot exceed the permissible value equal to 1/250 of the span between supports, according to [43], for the quasi-permanent load case. In addition, it was assumed that the floor is located in an office building and is subjected to the following loads, evenly distributed over the entire surface of the slab. One of them is the useful load with a characteristic value of: $q_{k}=3\;kN/m^{2}$ (according to [44] for office rooms). In addition, it was assumed that an equivalent load from partition walls equal to: $q_{k}=0.8\;kN/m^{2}$, and a permanent load outside the weight of the slab structure with a value of: $g_{k}=1.5\;kN/m^{2}$, are applied to the ceiling. The ceiling weight is a variable value and depends on the geometrical parameters of the slab and bubble cross-section, so it was directly taken into account in the cost function of the optimization algorithm. The deflection arrow was determined for the case of quasi-permanent loads in the serviceability limit state; therefore, the factors reducing the load values according to [44] were applied.

### 2.2. Numerical Homogenization of the Slab

In the following work, the method of numerical homogenization based on the equivalence of strain energy between the three-dimensional reference model and the simplified shell model was used [19,34,35,45,46,47,48]. The above method has already been adapted to prefabricated concrete slabs reinforced with spatial trusses [35] and reinforced slabs with voids, such as the bubble deck [34]. This approach uses the classical formulation of the displacement-based finite element method, extracting individual values for internal nodes—subscript “i”, and external nodes—subscript “e”:

(1) $$Ku=F\to \left[\begin{array}{cc} K_{ee} & K_{ei}\\ K_{ie} & K_{ii} \end{array}\right]\left[\begin{array}{c} u_{e}\\ u_{i} \end{array}\right]=\left[\begin{array}{c} F_{e}\\ 0 \end{array}\right].$$

where: K is the stiffness matrix, **u** is a displacement vector of nodes, and F is the external nodal load vector.

**Figure 2:**
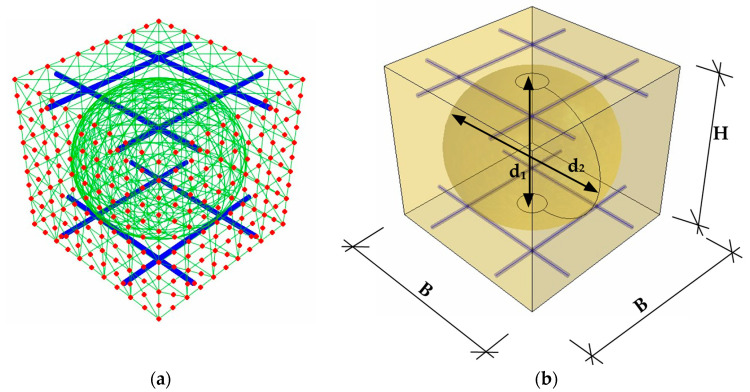
RVE: (**a**) external (in red color) and internal nodes and (**b**) parametrized for optimization purposes.

For this purpose, it is necessary to separate a representative volume element (RVE) from the model and perform static condensation. Condensation is the elimination of secondary degrees of freedom; in this case, internal nodes. Then, it is necessary to redefine the stiffness matrix with a reduced number of degrees of freedom only at the external nodes (see Figure 2a).

Additionally, the presented method of homogenization uses the relationship between the total energy of elastic deformation stored in the system after static condensation and the work of external forces on appropriate displacements:

(2) $$E=\frac{1}{2}u_{e}^{T}F_{e}$$

The homogenization method, as in [34,35], has been modified here to include only translational degrees of freedom (for two types of finite elements used in the models—truss and continuum). Therefore, the relationship between the generalized strain constants and the location of the external RVE nodes is expressed by the following transformation:

(3) $${\left[\begin{array}{c} u_{x}\\ u_{y}\\ u_{z} \end{array}\right]}_{i}={\left[\begin{array}{cccccccc} x & 0 & y/2 & z/2 & 0 & xz & 0 & yz/2\\ 0 & y & x/2 & 0 & z/2 & 0 & yz & xz/2\\ 0 & 0 & 0 & x/2 & y/2 & -x^{2}/2 & -y^{2}/2 & -xy/2 \end{array}\right]}_{i}{\left[\begin{array}{c} \varepsilon_{x}\\ \varepsilon_{y}\\ \gamma_{xy}\\ \gamma_{xz}\\ \gamma_{yz}\\ \kappa_{x}\\ \kappa_{y}\\ \kappa_{xy} \end{array}\right]}_{i},$$

Using the definition of the elastic strain energy for a discrete model:

(4) $$E=\frac{1}{2}u_{e}^{T}Ku_{e}=\frac{1}{2}\epsilon_{e}^{T}A_{e}^{T}KA_{e}\epsilon_{e.}$$

Taking into account the finite element model including bending, tension, and transverse shear, the elastic internal energy for the plate or shell can be expressed as:

(5) $$E=\frac{1}{2}\epsilon_{e}^{T}A_{k}\epsilon_{e}\{area\}$$

Thanks to this, the stiffness matrix for the homogenization method can be extracted from the discrete matrix:

(6) $$A_{k}=\frac{A_{e}^{T}KA_{e}}{area}.$$

Then, after appropriate transformations, we can obtain the stiffness matrix $A_{k}$, which is the ABDR matrix, consisting of all the required compression, bending, and shear stiffnesses:

(7) $$A_{k}=\left[\begin{array}{ccc} A_{3\times 3} & B_{3\times 3} & 0\\ B_{3\times 3} & D_{3\times 3} & 0\\ 0 & 0 & R_{2\times 2} \end{array}\right]$$

More information on numerical homogenization based on strain energy equivalence can be found in [19,34,35,45,46,47,48].

The design parameters, $\bar{x}$, that were used to determine the representative RVE for a specific design of the bubble bridge slab in the analyzed optimization problem are shown in Figure 2b. The assumed designed parameters read:

(8) $$\bar{x}=\{B,H,d_{1},d_{2}\}$$

in which B is the width and length of the RVE concrete unit, H is the height of the RVE concrete unit, and $d_{1},d_{2}$ are the dimensions of the ellipsoidal void, height, and horizontal diameter, respectively.

### 2.3. Study Framework and Optimization Problem Definition

In everyday challenges, structural engineers tackle various problems, and one of the most common is the optimal design of the structure. In this paper, the optimal design of the bubble deck slab in regard to not exceeding the serviceability limit state (SLS) and minimal use of the concrete is analyzed. The problem is not trivial since the bubble deck slabs have variable cross-sections. In regions with full concrete cross-sections, the plate has a higher stiffness, while for bubble void regions, the plate is less stiff. The properties of the plate periodically vary across the span, which makes it difficult to calculate the displacement field of such slab. Additionally, the minimal use of the concrete is opposed to limiting plate deflection. Therefore, the typical approach for computations must be extended in order to meet the requirements of the SLS and limit the use of material.

In this paper, the numerical homogenization technique was used to determine the effective bending stiffness for computing the plate displacement via the analytical formula. The homogenization technique used here was that presented by Garbowski and Gajewski [22]. The bending stiffnesses of $D_{11}$, $D_{22}$, $D_{12}$, and $D_{33}$ were determined by the homogenization technique in [22], which was used in multiple papers [34,45,46,47,48].

The square and symmetric bubble deck concrete slab was considered here, and the structure was reinforced with upper and lower steel mesh with ϕ10 steel bars. The cross-section design of the concrete bubble deck was described by a parametric RVE model with the design parameters gathered in $\bar{x}$ (see Section 2.2). The span dimensions of the slab were assumed to be $12\times 12\;m^{2}$, with evenly distributed load $q_{0}$. See Section 2.1.2 for more details of the assumed load.

The governing equation for the Kirchhoff–Love plate takes the following form [49,50]:

(9) $$D_{11}\frac{\partial^{4}w}{\partial x^{4}}+2\left(D_{12}+2D_{33}\right)\frac{\partial^{4}w}{\partial x^{2}\partial y^{2}}+D_{22}\frac{\partial^{4}w}{\partial y^{4}}=q.$$

in which w is the transverse deflection, x and y are the in-plane coordinates of the plate, and q is the transverse load.

The assumed plate was simply supported for all edges; therefore:

(10) $$a:\begin{array}{ccc} x_{1}=0, & w=0, & M_{1}=0 \end{array}.\;b:\begin{array}{ccc} x_{2}=0, & w=0, & M_{2}=0 \end{array}.$$

in which a, b are the dimensions of the floor slab (here, a=12 m and b=12 m were assumed), and $x_{1}$, $x_{2}$ are the orthogonal coordinates along the perpendicular edges, respectively.

It was assumed that the orthotropic plate was subjected to a transverse, uniformly distributed load, labeled as $q_{0}$:

(11) $$q\left(x_{1},x_{2}\right)=q_{0}=q_{0}\left(\bar{x}\right).$$

For more details regarding the determination of the uniformly distributed load, $q_{0}$, please refer to Section 2.1.2.

The final form of the plate deflection read:

(12) $$w\left(\bar{x}\right)=\frac{16q_{0}}{\pi^{6}}\sum_{m=1}^{\infty }\sum_{n=1}^{\infty }\frac{\sin\frac{m\pi x_{1}}{a}\sin\frac{n\pi x_{2}}{b}}{mn\left[D_{11}\left(\bar{x}\right){\left(\frac{m}{a}\right)}^{4}+2\left(D_{12}\left(\bar{x}\right)+2D_{33}\left(\bar{x}\right)\right){\left(\frac{mn}{ab}\right)}^{2}+D_{22}\left(\bar{x}\right){\left(\frac{n}{b}\right)}^{4}\right]}.$$

in which m,n are the odd numbers.

The total cost function, F, in the optimization problem to be solved takes the two following components:

(13) $$F\left(\bar{x}\right)=\omega F_{vol}\left(\bar{x}\right)+F_{defl}\left(\bar{x}\right).$$

in which $F_{vol}$ is responsible for decreasing the concrete use and $F_{defl}$ regards not exceeding the serviceability limit state due to the Eurocode standard [43]. The dimensionless factor ω is the scaling factor of the previous two and was selected by trial and error, with the aim of balancing the influence of both components on the objective function. Therefore, in this study, ω was set to $0.2\times 10^{-9}$. The mathematical details of the optimization algorithm used for minimizing the cost function, $F(\bar{x})$, were included in Section 2.4.

The total volume of the bubble deck floor slabs, $F_{vol}$, was calculated by using a single representative volume element of the bubble deck unit:

(14) $$V_{RVE}\left(\bar{x}\right)=B\cdot L\cdot H-\frac{4}{3}\pi \frac{d_{1}}{2}{\left(\frac{d_{2}}{2}\right)}^{2}.$$

Therefore, the first component of the cost function, $F_{vol}$, read:

(15) $$F_{vol}\left(\bar{x}\right)=\frac{ab}{BL}V_{RVE}\left(\bar{x}\right).$$

The second component of the cost function, $F_{defl}$, was computed based on the maximum slab deflection (computed by Equation (12)), combined with the serviceability limit state:

(16) $$F_{defl}\left(\bar{x}\right)=\left|w\left(\bar{x}\right)-\frac{\min\left(a,b\right)}{250}\right|.$$

In the optimization problem, the boundary limits of each design parameter of the concrete bubble deck were assumed and are presented in Table 1, where $b_{min}$ is the lower, while $b_{max}$ is the upper boundary of the physical dimensions. In Section 2.2, full details regarding the meaning of the symbols are presented, including exemplary graphics.

Since the dimensions of the bubble changed in the optimization process and the bubble was immersed in concrete with a variable height and width of the RVE module, physical inequality restrictions should be introduced. Therefore, the following inequality constraints were adopted:

(17) $$\begin{array}{c} d_{1}-H+40\leq 0\\ d_{2}-B+40\leq 0 \end{array}$$

In Equation (17), 40 represents the concrete bubble cover in mm that is, the distance between the surface of the bubble void and the outer surface of the concrete at cardinal points. The assumed nominal value of the concrete cover regarding the steel mesh was 35 mm.

Local search algorithms, such as the one used in this paper, are vulnerable to find the locally optimal solutions. Therefore, in order to minimize the probability that a globally and not locally optimal solution will be found, the optimization algorithm was run many times from different starting points (initial guesses) (see Table 2). This is a typical approach for better exploration of the multi-dimensional space of the design parameters.

All computations in the research, apart from computing the stiffness matrices of RVEs (see Section 2.2), were performed using MATLAB software (ver. 2023a) [51].

### 2.4. Mathematical Optimization Procedure

Among the many available methods of optimization, the sequential quadratic programming (SQP) method is one of the most reliable and trustworthy. This mainly regards its efficiency, namely, the smallest number of cost function evaluations is obtained in the benchmark examples and sufficient accuracy is maintained [52,53,54,55,56]. Therefore, in this study, the SQP method was used, as in [57].

The classical optimization problem read [56]:

(18) $$\min F(\bar{x}),$$

in which $F(\bar{x})$ is the cost function of the sought parameters, $\bar{x}$. The sough parameters may be constrained with equalities:

(19) $$\begin{array}{c} C_{eq}\left(\bar{x}\right)=0,\\ A_{eq}\cdot \bar{x}=b_{eq}, \end{array}$$

and/or more complex constraints may be more adequate. For instance, the nonequality constraints:

(20) $$\begin{array}{c} C\left(\bar{x}\right)\leq 0,\\ A\cdot \bar{x}\leq b,\\ b_{min}\leq \bar{x}\leq b_{max}, \end{array}$$

in which b, $b_{eq}$ are one-column matrices, A, $A_{eq}$ are matrices, C, $C_{eq}$ are functions, and $b_{min}$ and $b_{max}$ represent the lower and upper boundaries of the sought parameters, $\bar{x}$.

Constraints in the function $F(\bar{x})$ were computed by utilizing the Lagrange’s function approach, L. Thus, the mathematically equivalent subproblem is defined by the following:

(21) $$L\left(\bar{x},\lambda \right)=F\left(\bar{x}\right)+\sum_{i=1}^{m}\lambda_{i}\cdot g(\bar{x}),$$

in which $\lambda_{i}$ are the so-called Lagrange multipliers, while $g_{i}(\bar{x}$) are the constraints of nonequality.

In the SQP method, the following form of quadratic programming was solved:

(22) $$\underset{d\in R^{n}}{\min}\frac{1}{2}d^{T}H_{k}+\nabla F{\left({\bar{x}}_{k}\right)}^{T}d,$$

in which $H_{k}$ is the positive definite approximation of the Hessian matrix, which approximates Equation (21). The approximation of the Hessian matrix was modified at each primary iteration by the Broyden–Fletcher–Goldfarb–Shanno (BFGS) method:

(23) $$H_{k+1}=H_{k}+\frac{q_{k}q_{k}^{T}}{q_{k}^{T}s_{k}}-\frac{H_{k}s_{k}s_{k}^{T}H_{k}^{T}}{s_{k}^{T}H_{k}s_{k}},$$

in which:

(24) $$s_{k}={\bar{x}}_{k+1}-{\bar{x}}_{k},$$

(25) $$q_{k}=\left(\nabla F\left({\bar{x}}_{k+1}\right)+\sum_{i=1}^{m}\lambda_{i}\nabla g_{i}({\bar{x}}_{k+1})\right)-\left(\nabla F\left({\bar{x}}_{k}\right)+\sum_{i=1}^{m}\lambda_{i}\nabla g_{i}\left({\bar{x}}_{k}\right)\right).$$

A new step was computed based on the solution of the quadratic programming problem:

(26) $${\bar{x}}_{k+1}={\bar{x}}_{k}+\alpha_{k}d_{k},$$

in which $\alpha_{k}$ is the step length obtained by minimization of the objective function [52,53,54,55,56].

## 3. Results

In local search optimization algorithms, it is recommended to solve multiple optimization problems to determine the solution, which is not locally but globally optimal. Therefore, the optimization procedure was conducted for several initial guesses of the design parameters to find the best solution, and the solutions of the initial assumed guesses were presented in Section 2.3. The results obtained from solving the optimization problem stated in Section 2.3 by the optimization method shown in Section 2.4 are summarized in Table 3. The second to fifth columns present the optimal parameters of the concrete bubble deck designs. In column six, the slab deflection obtained for the optimal designs due to the uniformly distributed load can be found. Moreover, the seventh to ninth columns show the components of the cost function and the total value of the cost function.

**Figure 3:**
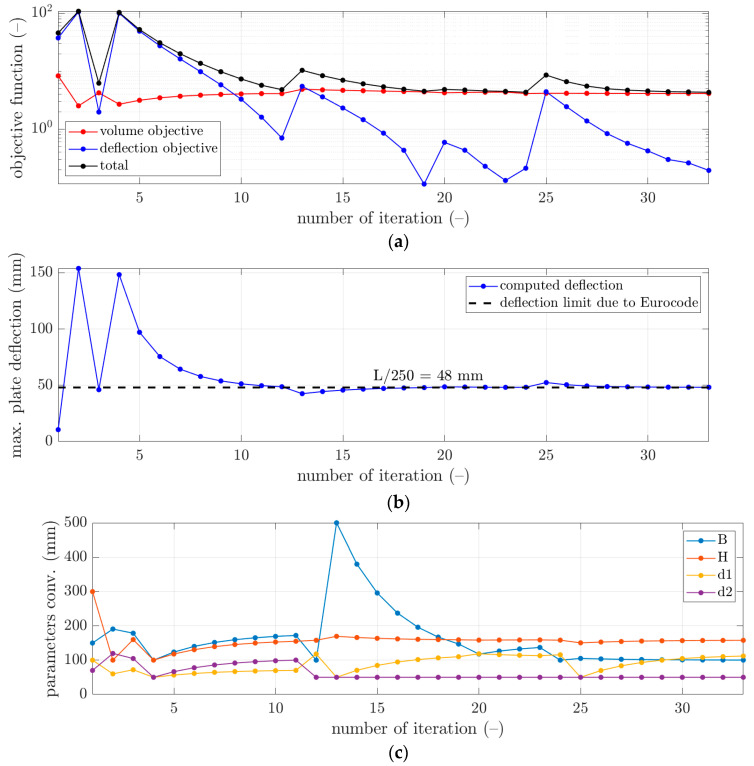
The convergence of the optimal selection of geometrical parameters of the bubble deck slab for initial guess ${{\bar{x}}^{0}}_{1}$: (**a**) cost function, (**b**) verification of the serviceability limit state, and (**c**) derived bubble deck parameters.

**Figure 4:**
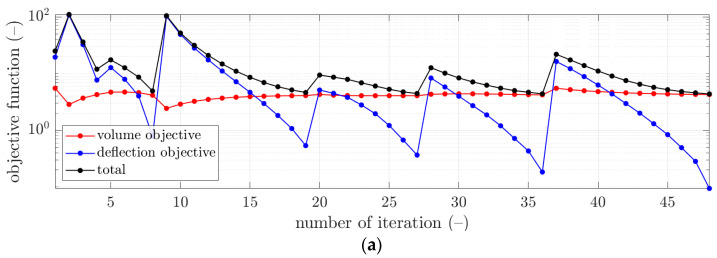

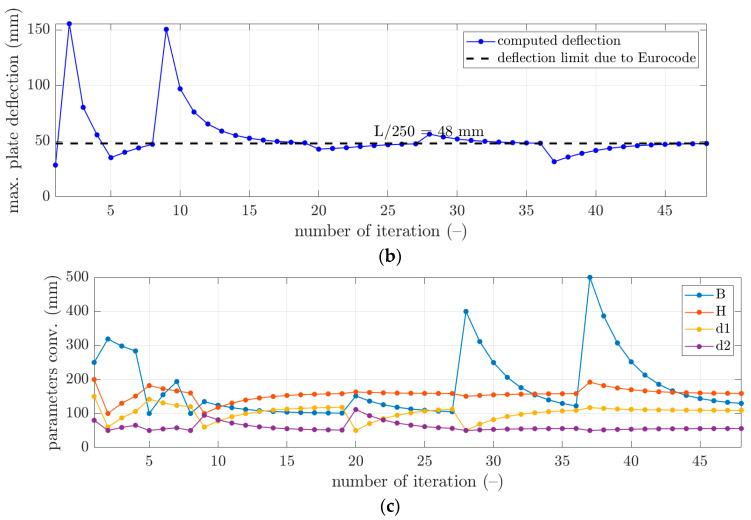
The convergence of the optimal selection of geometrical parameters of the bubble deck slab for initial guess ${{\bar{x}}^{0}}_{2}$: (**a**) cost function, (**b**) verification of the serviceability limit state, and (**c**) derived bubble deck parameters.

**Figure 5:**
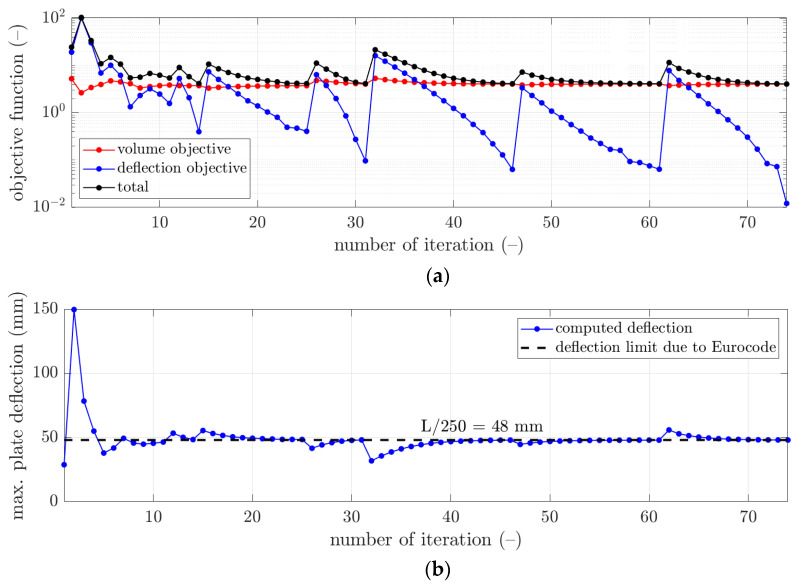

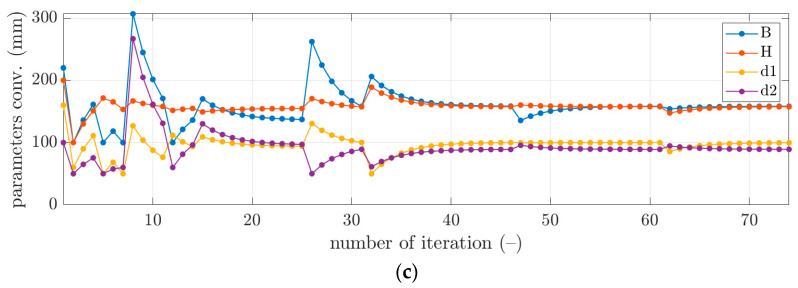
The convergence of the optimal selection of geometrical parameters of the bubble deck slab for initial guess ${{\bar{x}}^{0}}_{3}$: (**a**) cost function, (**b**) verification of the serviceability limit state, and (**c**) derived bubble deck parameters.

More details of the convergence of the solutions are presented for selected examples from Table 3 in Figure 3, Figure 4 and Figure 5. In each figure, the minimization of the cost function, F, is demonstrated with its components for iterations of the optimization algorithm, i.e., $\omega F_{vol}$—component of minimizing the volume of the concrete, and $F_{defl}$—component of minimizing the maximum plate deflection (see Figure 3a, Figure 4a and Figure 5a). Additionally, in Figure 3b, Figure 4b and Figure 5b, the maximum plate deflection was confronted with the SLS condition from the Eurocode standard [43]. For the analyzed case of the slab, namely, $12\times 12\;m^{2}$, the limit computed from the 1/250 condition was equal to 48 mm. In Figure 3b, Figure 4b and Figure 5b, the limit is marked with a dashed line. In addition, in Figure 3c, Figure 4c and Figure 5c, the changes of the sought parameters of B, H, $d_{1}$, and $d_{2}$ within the optimization are shown, showing the convergence to the final sought parameters of the bubble deck slab.

## 4. Discussion

The optimal design of the bubble deck slab floor regarding concrete use and the SLS is not a trivial task. The main difficulty is determining the mechanical properties of the periodically changing cross-section of the plate. Fully detailed finite element modeling of such structures is time-consuming in modeling and computations. Therefore, for this reason, for engineering purposes, the method presented in the paper is highly attractive. It does not require full formal finite element analysis of the floor slab, but only building the global stiffness matrix of the single periodic RVE unit and straightforward post-computations to obtain effective stiffnesses.

Therefore, in this paper, the complex structure of the bubble deck slab was considered to determine the optimal solution without using a typical, less accurate method. In this paper, the minimization of $F_{vol}$ and $F_{defl}$ components was in contrast; therefore, it was typical that the $F_{vol}$ component decreased, while the $F_{defl}$ component increased (for instance, see iterations 2 and 9 in Figure 4a). The rapid increase of the deflection plots was related to the principle of operation of the optimization algorithm, which changed the slab parameters so that the concrete volume was reduced. This increased the deflection, which, when L/250 was exceeded, activated a kind of penalty function ($F_{defl}$) that increased the total value of the objective function.

However, as presented in the optimization summary in Section 3, it was possible to obtain the deflection very close to the design standard limit, i.e., 48 mm. As shown in Table 3, all differences in the deflections achieved in relation to the design standard limit were approximately not bigger than 0.15 mm. Therefore, those components in the cost function outcomes were relatively small, not bigger than 0.12; however, the smallest was computed for the initial guess of ${{\bar{x}}^{0}}_{3}$, i.e., 0.0121. In Figure 3b, Figure 4b and Figure 5b, it can be observed that occasionally, the optimization algorithm broke the deflection limitation (for instance, see iterations 2 and 4 in Figure 3b), but it returned to respecting the limit after one or a few iterations. Similar features are visible in Figure 4b, iterations 2 and 9, and in Figure 5b, iterations 2, 12, and 15.

On the other hand, it was observed that the component related to the minimization of the concrete use yielded much greater values, that is, between 4.06 and 4.25. Since this component is the scaled volume of the concrete of the slab, it cannot be minimized to 0. Still, significant decreases of this component were observed compared to the initial guess values (see red plots in Figure 3a, Figure 4a and Figure 5a). Here, the lowest value was obtained for the initial guess of ${{\bar{x}}^{0}}_{3}$, i.e., 4.0593.

The best solution, that is, the globally optimal solution, was obtained for the initial guess of ${{\bar{x}}^{0}}_{3}$. Compared to the initial design of the bubble deck, the weight loss of the concrete was 23% (4.06, compared to 5.26 of $F_{vol}$). Therefore, the optimal parameters of the simply supported bubble deck slab of $12\times 12\;m^{2}$ for the uniformly distributed load were: B=158.2 mm, H=157.5 mm, $d_{1}=$ 99.7 mm, and $d_{2}=89.1\;mm$. Optimal parameter H was similar for all locally optimal solutions, as can be observed in Table 3, where it changed from 155.6 mm to 158.6 mm.

The main advantage of the methodology shown in the paper is the computational time of the analysis. The single evaluation of the cost function lasted less than 15 s. Therefore, in less than approximately 20 min, the single optimization procedure was finished. Going further, after about 2 h, the reasonable exploration of the design space can be achieved, and finally, the globally optimal solution can be expected.

## 5. Conclusions

The main aim of this paper was to find the optimal designs of the bubble deck slab subjected to a uniformly distributed load with regard to minimal concrete use and not exceeding the serviceability limit state of the Eurocode standard. In the research study, the numerical homogenization technique was used to determine the effective properties of the bubble deck slab within the cost function. Moreover, the local search algorithm of sequential quadratic programming was used in the minimization problem with linear constraints to derive the bubble deck slab module; that is, its length/width and height, but also the geometry of the ellipsoidal bubble void, i.e., its height and horizontal diameter.

As confirmed in the research, the optimization allowed to determine designs of the bubble deck slabs that ensured the minimum mass of concrete and met the serviceability limit state. Compared to the initial design of the bubble deck, the weight loss of the concrete was 23%. It was shown that the homogenization method used in this paper is highly attractive because it does not require solving complex structural problems through the computationally expensive finite element method. Achieving an optimal bubble deck design from a computational point of view would be carried out in just a few hours, instead of the heavy calculations of a single design case that would take the same amount of time. Due to the method used, the complex structure of the bubble deck slab was considered without using the typical method of substituting the cross-section of the concrete, which is less accurate due to shape simplification.

## Figures and Tables

**Table 1:** The lower and upper boundary values of the parameters selected for optimization of the concrete part of the bubble deck slab.

Boundary	B (mm)	H (mm)	$d_{1}$ (mm)	$d_{2}$ (mm)
$b_{min}$	100	100	50	50
$b_{max}$	500	500	500	500

**Table 2:** The initial guesses of the design parameters selected for optimization of the concrete part of the bubble deck slab.

No.	B (mm)	H (mm)	$d_{1}$ (mm)	$d_{2}$ (mm)
${{\bar{x}}^{0}}_{1}$	150	300	100	70
${{\bar{x}}^{0}}_{2}$	250	200	150	80
${{\bar{x}}^{0}}_{3}$	220	200	160	100
${{\bar{x}}^{0}}_{4}$	200	250	110	180
${{\bar{x}}^{0}}_{5}$	170	150	90	90

**Table 3:** Optimal designs of the concrete bubble deck slab with corresponding cost function values obtained by the optimization algorithm.

No.	B (mm)	H (mm)	$d_{1}$ (mm)	$d_{2}$ (mm)	w (mm)	$\omega F_{vol}$ (–)	$F_{defl}$ (–)	F (–)
${{\bar{x}}^{0}}_{1}$	100.0	158.6	115.8	50.0	47.94	4.1314	0.0616	4.1930
${{\bar{x}}^{0}}_{2}$	122.1	158.5	109.1	55.9	48.02	4.2193	0.0164	4.2357
${{\bar{x}}^{0}}_{3}$	158.2	157.5	99.7	89.1	48.01	4.0593	0.0121	4.0714
${{\bar{x}}^{0}}_{4}$	110.7	156.0	52.8	70.5	48.12	4.2505	0.1165	4.3669
${{\bar{x}}^{0}}_{5}$	144.8	155.6	53.8	104.4	48.04	4.0600	0.0390	4.0990

## Data Availability

The data presented in this study are available upon request from the corresponding author.
